# The PI3Kδ-Selective Inhibitor Idelalisib Minimally Interferes with Immune Effector Function Mediated by Rituximab or Obinutuzumab and Significantly Augments B Cell Depletion In Vivo

**DOI:** 10.4049/jimmunol.1700323

**Published:** 2018-02-16

**Authors:** Adam Palazzo, Sylvia Herter, Laura Grosmaire, Randy Jones, Christian R. Frey, Florian Limani, Marina Bacac, Pablo Umana, Robert J. Oldham, Michael J. E. Marshall, Kerry L. Cox, Anna H. Turaj, Mark S. Cragg, Christian Klein, Matthew J. Carter, Stacey Tannheimer

**Affiliations:** * Gilead Sciences, Inc., Foster City, CA 98102;; † Roche Pharmaceutical Research and Early Development, Roche Innovation Center Zurich, CH-8952 Zurich, Switzerland; and; ‡ Antibody and Vaccine Group, Cancer Sciences Unit, Faculty of Medicine, University of Southampton, Southampton SO17 1BJ, United Kingdom

## Abstract

Idelalisib is a highly selective oral inhibitor of PI3Kδ indicated for the treatment of patients with relapsed chronic lymphocytic leukemia in combination with rituximab. Despite additive clinical effects, previous studies have paradoxically demonstrated that targeted therapies potentially negatively affect anti-CD20 mAb effector mechanisms. To address these potential effects, we investigated the impact of PI3Kδ inhibition by idelalisib on the effector mechanisms of rituximab and obinutuzumab. At clinically relevant concentrations, idelalisib minimally influenced rituximab- and obinutuzumab-mediated Ab-dependent cellular cytotoxicity and phagocytosis on human lymphoma cell lines, while maintaining the superiority of obinutuzumab-mediated Ab-dependent cellular cytotoxicity. Consistent with this, idelalisib did not influence obinutuzumab-mediated B cell depletion in whole-blood B cell–depletion assays. Further, idelalisib significantly enhanced obinutuzumab-mediated direct cell death of chronic lymphocytic leukemia cells. In murine systems, in vivo inhibition of PI3Kδ minimally interfered with maximal rituximab- or obinutuzumab-mediated depletion of leukemic targets. In addition, the duration of rituximab- and obinutuzumab-mediated depletion of leukemia cells was extended by combination with PI3Kδ inhibition. Collectively, these data demonstrate that PI3Kδ inhibition does not significantly affect the effector mechanisms induced by rituximab or obinutuzumab and provides an effective in vivo therapeutic combination. Therefore, combinations of obinutuzumab and idelalisib are currently being assessed in clinical studies.

## Introduction

Phosphatidylinositol 3-kinase δ represents the most prominent PI3K isoform in B lymphocytes. As such, PI3Kδ is central to multiple signaling pathways that drive the proliferation, survival, homing, and retention of malignant B cells within primary and secondary lymphoid organs. Accordingly, PI3Kδ represents a prime target for therapeutic intervention in B cell malignancies and is effectively targeted by idelalisib, a highly selective oral inhibitor of PI3Kδ (1, 2). Idelalisib functions by selective prevention of ATP binding to the catalytic domain of PI3Kδ, thereby preventing phosphorylation of phosphatidylinositol and subsequent serine/threonine protein kinase B phosphorylation (3). In the United States, idelalisib is indicated, in combination with rituximab, for the treatment of patients with relapsed chronic lymphocytic leukemia (CLL) and as monotherapy for relapsed follicular B cell non-Hodgkin lymphoma (FL) and relapsed small lymphocytic lymphoma (4). In the European Union, idelalisib is indicated, in combination with rituximab or ofatumumab, for the treatment of patients with relapsed CLL, as first-line therapy in CLL patients with the 17p deletion or *TP53* mutation who are deemed unsuitable for chemoimmunotherapy, and as monotherapy for patients with refractory FL (5).

Type I anti-CD20 mAbs, such as rituximab, rapidly induce the redistribution of CD20 within the plasma membrane to a low-density detergent-insoluble membrane compartment, which may affect binding properties and effector functions that control the therapeutic effect of anti-CD20 mAbs (6, 7). In contrast, type II anti-CD20 mAbs (such as obinutuzumab) do not induce significant CD20 redistribution and, as such, impart enhanced therapeutic effects, including direct killing of cellular targets by homotypic adhesion (7–9). In addition to its type II properties, obinutuzumab is glycoengineered and consequently offers enhanced affinity for FcγRIII and increased Ab-dependent cellular cytotoxicity (ADCC) and Ab-dependent cellular phagocytosis (ADCP) in comparison with rituximab (10, 11).

Obinutuzumab has been approved for first-line treatment of CLL patients in combination with chlorambucil in the United States and Europe and for first-line treatment of FL in Europe, based on head-to-head trials comparing obinutuzumab regimens with the respective rituximab regimen using a flat dose of 1000 mg for obinutuzumab and 375 mg/m^2^ for rituximab, as well as for the treatment of rituximab-refractory FL patients (12–15). In first-line diffuse large B cell lymphoma, obinutuzumab did not show superior outcomes (16, 17).

Because anti-CD20 mAbs are the standard of care, it is important to understand whether new targeted agents affect their function. Previous work has shown that the covalent Bruton’s tyrosine kinase inhibitor, ibrutinib, can interfere with immune effector function and, ultimately, with in vivo efficacy of rituximab in preclinical models (18). Because PI3K isoforms also play a role in immune effector cells and FcγR signaling (19), we investigated the effect of PI3Kδ inhibition by idelalisib on the immune effector functions of rituximab and obinutuzumab and the efficacy of in vivo anti-CD20 mAb therapy in a murine model of CLL.

## Materials and Methods

### Reagents and chemicals

Idelalisib was synthesized at Gilead Sciences, dissolved in DMSO at 10 mM, and stored at −20°C. Rituximab and obinutuzumab were provided by Hoffmann–La Roche (Basel, Switzerland). Palivizumab was used as a negative control and was produced at Gilead Sciences.

### Cell culture

WIL2-S cells were obtained from the American Type Culture Collection (Manassas, VA) and maintained in IMDM supplemented with 10% ultra-low Ig FBS and 1% penicillin-streptomycin (all from Life Technologies [Thermo Fisher Scientific], Grand Island, NY).

For macrophage polarization, frozen CD14^+^ monocytes enriched by negative selection were thawed and cultured in T75 tissue flasks in AIM-V medium (Life Technologies) with 60 ng/ml M-CSF (PeproTech, Rocky Hill, NJ). On day 7, monocyte-derived macrophages (MDMs) were washed and plated in AIM-V with polarizing cytokines. For differentiation to M1 macrophages, cells were plated for 24 h in 100 ng/ml IFN-γ (R&D Systems, Minneapolis, MN) and 100 ng/ml LPS (derived from *Escherichia coli* strain 055:B5; Sigma-Aldrich); for differentiation to M2c macrophages, cells were plated for 48 h in 10 ng/ml IL-10 (R&D Systems).

### ADCC assay with PBMC effectors

PBMCs were prepared by Histopaque (Sigma-Aldrich) density centrifugation of fresh blood obtained from healthy human donors. WIL2-S target cells (2.5 × 10^4^ cells per well) were incubated with isolated human PBMCs (6.25 × 10^5^ cells per well), as well as with titrations of obinutuzumab or rituximab (0.01–1000 ng/ml) in the presence or absence of 256 nM idelalisib in AIM-V medium for 4 h in a humidified incubator at 37°C, 5% CO_2_. Ten microliters per well of a 1:10 predilution of anti-CD107a–PE Ab (catalog number 328608; BioLegend, San Diego, CA) was added into the culture to determine NK cell degranulation. Tumor cell lysis was assessed after 4 h of incubation at 37°C, 5% CO_2_ by quantification of lactate dehydrogenase (LDH) released into cell culture supernatants (LDH detection kit, catalog number 11644793001; Roche Applied Science, Indianapolis, IN). Maximal lysis of the target cells (= 100%) was achieved by incubation of target cells with 2% Triton X-100. Spontaneous release (= 0%) refers to target cells coincubated with effector cells without Abs. ADCC was calculated using the following formula:

$$\text{Percentage ADCC}=\left(\left[\frac{\text{sample release}-\text{spontaneous release}}{\text{maximal release}-\text{spontaneous release}}\right]\right)\times 100.$$

For the assessment of NK cell degranulation, surface staining for CD3 (PE-Cy7, anti-human CD3 Ab, catalog number 300420) and CD56 (allophycocyanin anti-human CD56, catalog number 318310; both from BioLegend) was performed. After a 30-min incubation at 4°C in the dark, cells were washed twice with 150 μl per well of PBS/0.1% BSA and fixed using 100 μl per well of BD FACS Lysing Solution (catalog number 349202; BD Biosciences). Samples were analyzed using a BD FACSCanto II.

### ADCC assay with NK effectors

PBMCs from healthy donors were isolated by standard Ficoll density gradient centrifugation technique, and NK cells were enriched using a negative-selection immunomagnetic enrichment kit (STEMCELL Technologies, Vancouver, BC, Canada), according to the manufacturer’s instructions. Enriched NK cells were incubated overnight in RPMI 1640 with GlutaMAX supplemented with 10% ultra-low Ig FBS and 1% penicillin-streptomycin at 37°C in a 5% CO_2_ incubator before NK cells were combined with WIL2-S cells for ADCC assays. WIL2-S cells were resuspended at 2 × 10^6^ cells per milliliter in ADCC assay media consisting of RPMI 1640 Medium, no phenol red (Life Technologies), supplemented with 1% low Ig FBS (Life Technologies) and 1% penicillin-streptomycin. Target cells (WIL2-S) were plated in 96-well deep-well plates in triplicate. Idelalisib or 0.2% DMSO was added to target cells and incubated for 40 min at 37°C in 5% CO_2_. Obinutuzumab, rituximab, or palivizumab was prepared in ADCC assay media. After incubation for 40 min in compound or vehicle, each Ab was added to target cells and incubated at room temperature for 20 min before ADCC assay setup. Effector cells (enriched NK cells) were incubated with idelalisib or 0.2% DMSO and incubated for 1 h at 37°C, 5% CO_2_. Effector cells were added to wells containing target cells to reach E:T ratios of 1:1 to 30:1, comparable to those used by Kohrt et al. (18). Control wells included target cells with mAbs, in combination or not with idelalisib, as well as target cells alone. Additionally, effector cells in the presence or absence of idelalisib were plated as controls. The plates were incubated for 4 h at 37°C in a 5% CO_2_ incubator. LDH release was measured using a cytotoxicity detection kit (Roche Applied Science), following the manufacturer’s instructions. Absorbance values were measured using a SpectraMax Microplate Reader (Molecular Devices, Sunnyvale, CA) set at 490 nm, with a reference wavelength at 650 nm. ADCC was calculated using the previously described formula. The percentage of maximum was calculated using the following formula:

100−([(normal activity−inhibited activity)÷(normal activity)]×100)

with normal activity being the percentage of ADCC without the addition of idelalisib.

### ADCC assay with CLL targets

Frozen CLL PBMCs were thawed and washed in medium before resuspending in PBS (Severn Biotech) at 1 × 10^7^ cells per milliliter. Ten microliters of Calcein, AM (1 mg/ml; Life Technologies) was added per milliliter, and cells were incubated at 37°C in 5% CO_2_ for 30 min with periodic mixing. Cells were washed three times (300 × g, 5 min) with PBS (10% FCS) and resuspended in medium at 9 × 10^5^ cells per milliliter. Effector cells (PBMCs) from healthy donors were isolated from anonymized leukocyte cones (University of Southampton Tissue Bank, Southampton, U.K.) using Lymphoprep (AXIS-SHIELD) density gradient centrifugation and resuspended in medium at 20 × 10^6^ cells per milliliter. Target and effector cells were preincubated separately without treatment or with vehicle (DMSO) or with idelalisib (1 μM–1 nM) for 40 min at 37°C in 5% CO_2_. A total of 90 μl of target cells was transferred to a round-bottom 96-well plate (Thermo Fisher Scientific), and Ab was added to a final concentration of 10 μg/ml. Cells were incubated for an additional 20 min, after which 100 μl of effector cells with matching pretreatment was added (E:T ratio, 25:1), and the plate was briefly pulse centrifuged and incubated for 4 h at 37°C, 5% CO_2_. After incubation, the plate was centrifuged at 200 × *g* for 5 min, and 90 μl of supernatant was transferred to a white-walled 96-well plate. Calcein release was measured using a Varioskan plate reader (excitation 494 nM, emission 515 nM; Thermo Fisher Scientific), and lysis was calculated as a percentage of maximum lysis (4% Triton X-100) after subtraction of background (target cells plus effector cells without Ab).

### ADCP assay

Idelalisib was added to plated macrophages in AIM-V media and incubated at 37°C for 1 h, followed by the addition of rituximab or obinutuzumab. WIL2-S target cells were labeled with CellTracker Red (CTR; Molecular Probes; Thermo Fisher Scientific), as per the manufacturer’s protocol, and combined with the macrophages at an E:T ratio of 3:1. The cocultures were incubated for 2 h at 37°C. Cells were then stained with FITC anti-CD14 (BD Biosciences) and FITC anti-CD11b (eBioscience) and analyzed on an LSR II cytometer (BD Biosciences).

Cells within the live cell gate containing CD14^+^ M1 or M2c macrophages and CTR^+^ target cells were further separated within a dot plot quadrant. FITC^+^ effector macrophages were placed in the upper left quadrant of the *y*-axis, and CTR^+^ target cells were placed in the lower right quadrant of the *x*-axis. Double-positive cells (FITC^+^CTR^+^) in the upper right quadrant represent phagocytized target cells, and the percentage of phagocytosis was calculated as (% double-positive cells + [% double-positive cells + % target cells alone]) × 100.

### B cell depletion in human whole blood

Normal B cell depletion was also assessed using fresh heparinized human blood from healthy volunteers. Briefly, fresh blood was collected in heparin-containing syringes. Blood aliquots (180 μl per well) were placed into 96-well deep-well plates, supplemented with 10 μg/ml obinutuzumab or rituximab (10 μl per well) in the absence or presence of idelalisib, and incubated for 1 d at 37°C in 5% CO_2_ in a humidified cell incubator. After incubation, blood was mixed by pipetting up and down before blood aliquots (35 μl per well) were transferred into 96-well round-bottom plates and incubated with fluorescent anti-CD45 (anti-human CD45 allophycocyanin, catalog number 304037), anti-CD19 (anti-human CD19 PE, catalog number 302208), and anti-CD3 (anti-human CD3 PE-Cy7, catalog number 300420; all from BioLegend). After a 15-min incubation at room temperature, BD FACS Lysing Solution (BD Biosciences) was added to lyse erythrocytes and to fix cells prior to flow cytometry. B cell depletion was evaluated using samples not treated with Ab as a 100% control and the following formula:

$$100-\left(\left[\frac{100}{\text{B/T cell ratio in control}}\right]\right)\times \left(\left[\text{B/T cell ratio in sample containing Ab}\right]\right).$$

The average B cell depletion and SDs of the triplicates of each experiment were calculated.

### CLL cell death assay

Whole blood from CLL subjects was obtained through Bioreclamation (Westbury, NY), and PBMCs were collected by Ficoll separation and cryopreserved in freezing medium (50% IMDM, 40% FBS, and 10% DMSO). Frozen primary CLL PBMCs were thawed, washed, and rested for 3–5 h in RPMI 1640, 10 mM HEPES (pH 7.4), 1 mM sodium pyruvate, 2 mM GlutaMAX, 55 μM 2-ME, 100 U/ml penicillin/streptomycin, and 10% FBS at 37°C prior to plating. Cells were then plated at 1.0–2.5 × 10^6^ cells per milliliter in combination with idelalisib and Abs. Assay plates were incubated for 1 h prior to stimulation with anti-IgM/anti-IgG (20 μg/ml) and anti-CD40 (20 μg/ml). Cells were incubated at 37°C with 5% CO_2_ for 66 h, transferred to a deep-well plate, washed with 1 ml of cation-free PBS (PBS^−/−^), and resuspended in Live/Dead Fixable Aqua Dead Cell Stain (Life Technologies), according to the manufacturer’s instructions. After viability staining, cells were labeled with CD5-PE, CD19-BV421, and annexin V–allophycocyanin, washed, fixed with 4% paraformaldehyde, and acquired on a FACSCanto II flow cytometer (BD Biosciences). CD5^+^CD19^+^ cells were identified and gated for analysis. The CD5^+^CD19^+^ population was plotted as annexin V–allophycocyanin versus live/dead. Quadrant gates were drawn: Q1 is annexin V^−^ and live/dead^+^, Q2 is annexin V^+^ and live/dead^+^, Q3 is annexin V^+^ and live/dead^−^, and Q4 is double negative. Results are reported as the sum of Q1, Q2, and Q3 as a percentage of the total cells.

### Animals

Mice were maintained in local facilities, and experiments were approved by local ethical committees under UK Home Office license PPL number PB24EEE31. Eμ-TCL1–transgenic (Tg) mice were obtained from Dr. Egle (Salzburg Cancer Research Institute, Salzburg, Austria) with the consent of Dr. Pekarsky and Prof. Croce (Ohio State University). Eμ-TCL1 Tg animals were crossed with human CD20-Tg C57BL/6 mice (a kind gift from Prof. Shlomchik, University of Pittsburgh School of Medicine, Pittsburgh, PA), described previously by Ahuja et al. (20), and monitored for disease presentation. Terminal animals were sacrificed, and splenocytes were harvested and cryopreserved. Sex-matched C57BL/6 or SCID syndrome mice were inoculated with 1 × 10^7^ splenocytes derived from Eμ-TCL1–Tg or CD20-Tg Eμ-TCL1–Tg mice, respectively, and monitored for disease presentation by weekly blood sampling and flow cytometry. Following disease presentation, mice were treated with 250 μg of anti-mouse CD20 (clone 18B12 mouse IgG2a), 250 μg of rituximab (human IgG1 [hIgG1]), or 250 μg of obinutuzumab (hIgG1) by i.p. injection, together with 10 mg/kg GS-9820 by mouth twice a day or an appropriate vehicle control, as described previously (21). Disease progression/therapeutic responses were subsequently monitored by blood sampling and flow cytometry.

## Results

### Impact of idelalisib on immune effector functions of rituximab and obinutuzumab

**FIGURE 1.**
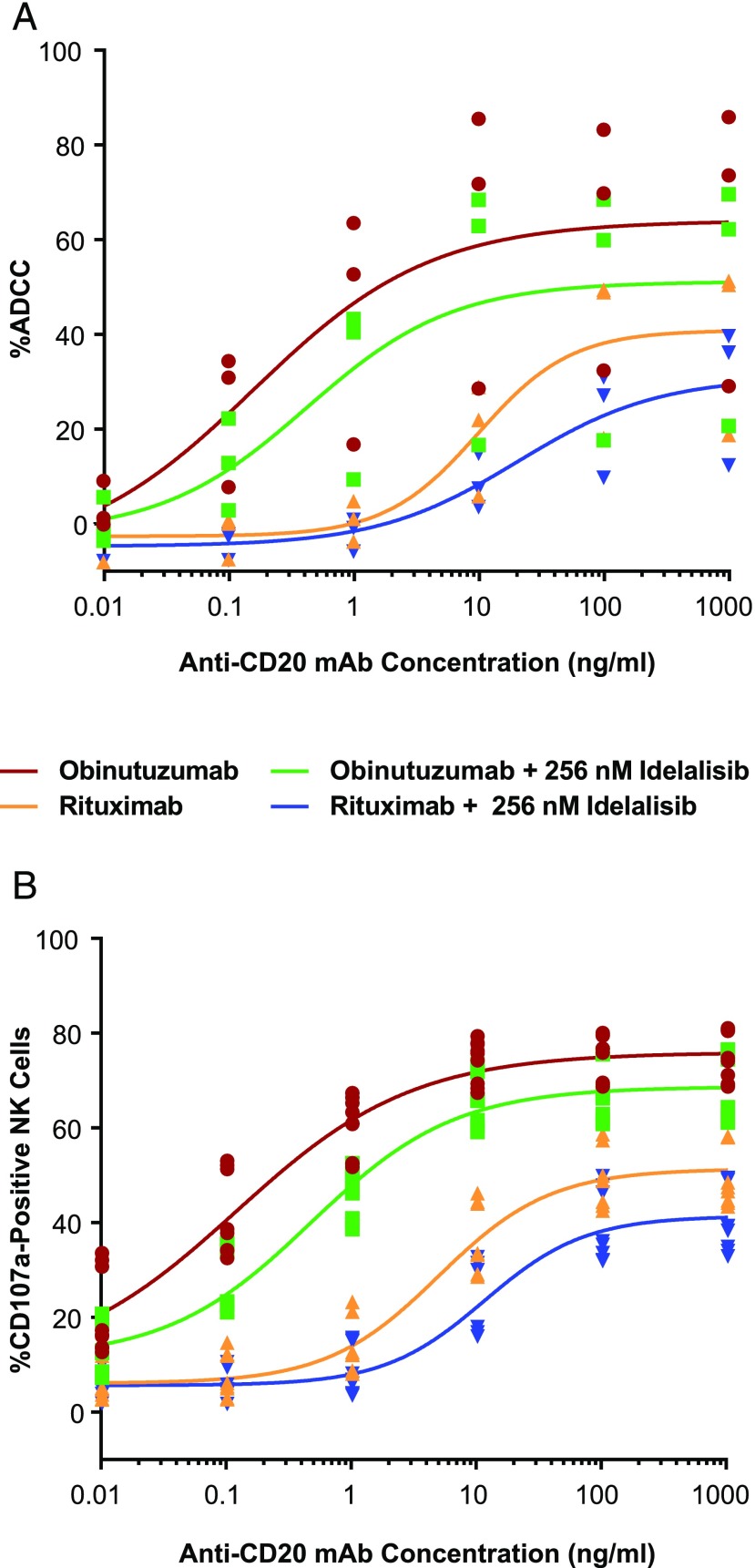
Effect of idelalisib on anti-CD20–mediated ADCC using healthy donor PBMCs. (**A**) Human PBMCs were used as effectors with the WIL2-S cell line at an E:T ratio of 25:1. Anti-CD20 mAb potency was assessed by LDH release after 4 h in the presence or absence of idelalisib (256 nM) over a range of Ab concentrations (0.01–1000 ng/ml). The mean of the triplicates for each of the three donors is shown as a single point (*n* = 3, in triplicate). (**B**) PBMCs were analyzed from the ADCC assay for the percentage of CD107a^+^ cells by gating on the CD3^−^CD56^intermediate^ population (NK cells), and frequency was determined for CD107a by flow cytometry as a measure of NK cell degranulation. The mean of the triplicates for each of the four donors is shown as a single point (*n* = 4, in triplicate).

**FIGURE 2.**
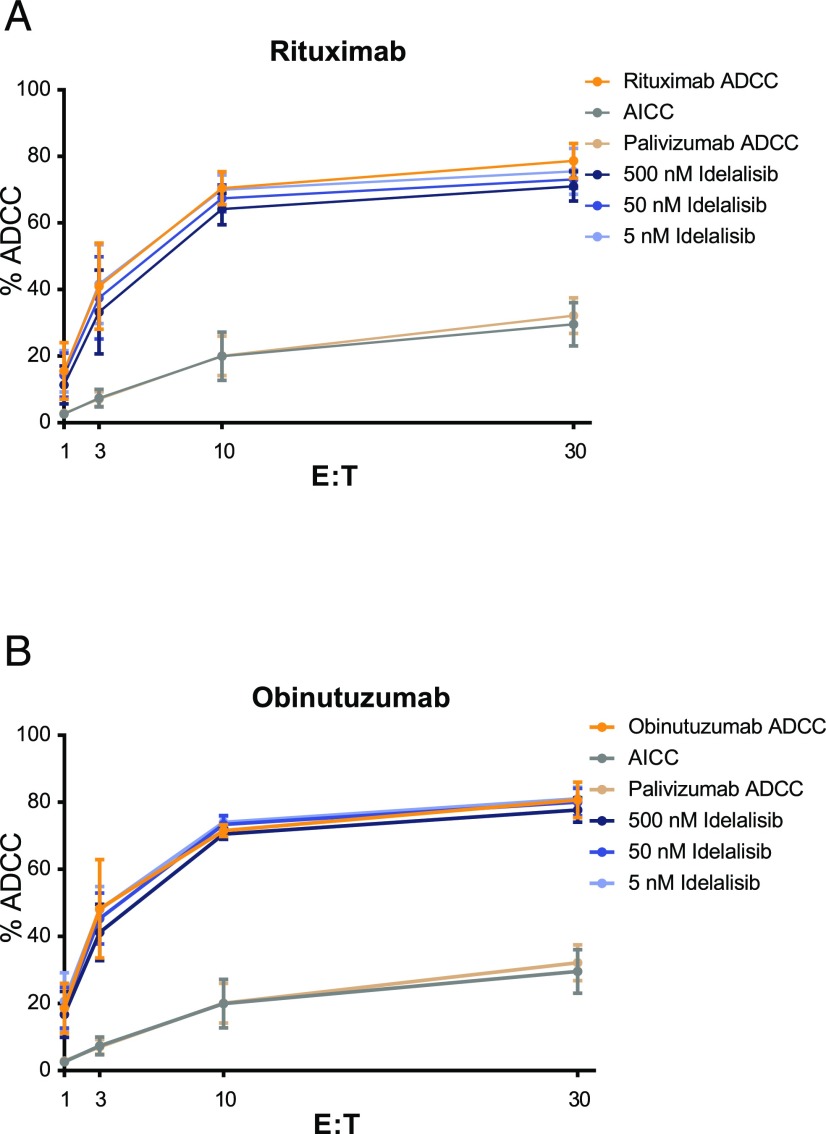
Idelalisib did not have an effect on ADCC mediated by rituximab (**A**) or obinutuzumab (**B**) with healthy donor NK cells. Enriched NK cells were used as effectors with the WIL2-S cell line as target over various E:T ratios. Anti-CD20 mAb potency was assessed at a saturating concentration (10 μg/ml) in the presence or absence of idelalisib (5–500 nM) by LDH release after 4 h (*n* = 3, in triplicate, mean ± SD). Palivizumab was used as a negative control Ab. NK cells and WIL2-S cells were incubated without Ab as an Ab-independent cellular cytotoxicity (AICC) control.

The potential impact of idelalisib on the immune effector functions of rituximab and obinutuzumab was first evaluated by measuring ADCC against the B cell lymphoma line WIL2-S in primary PBMC cultures with titrated amounts of Ab, in the presence or absence of a single concentration of idelalisib, representative of the cell culture medium protein-binding–adjusted maximum peak concentration (C_max_) of 256 nM. As expected, the magnitude of ADCC mediated by obinutuzumab was greater than that by rituximab, and the addition of idelalisib showed little to no effect on ADCC with either Ab (Fig. 1A). Effector NK cell surface expression analysis by flow cytometry showed a concentration-dependent increase in the frequency of CD107a^+^ NK cells with rituximab and obinutuzumab, with obinutuzumab inducing more profound effects (Fig. 1B). Addition of idelalisib had little effect on the frequency of CD107a^+^ cells or the density of surface CD107a (data not shown) with obinutuzumab or rituximab. When ADCC was evaluated over various E:T ratios using isolated NK cells (effectors) and WIL2-S cells (targets) and at saturating concentrations of rituximab and obinutuzumab (10 μg/ml), idelalisib had no effect on ADCC when tested over a dose range from 5 to 500 nM (Fig. 2).

**FIGURE 3.**
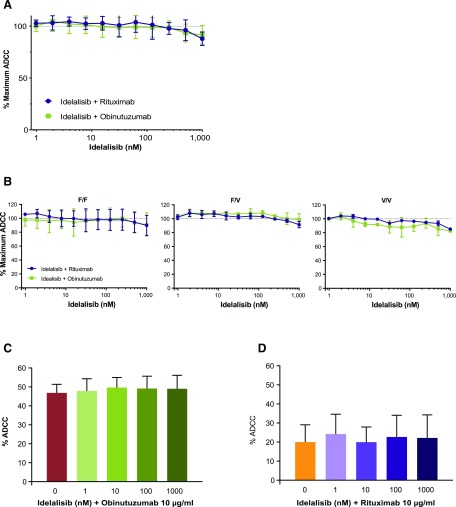
Effect of idelalisib on anti-CD20–mediated ADCC: impact of effectors, targets, and FcγRIIIa genotype. (**A**) Enriched NK cells were used as effectors with the WIL2-S cell line (E:T ratio, 10:1) and saturating anti-CD20 mAb (10 μg/ml), with the maximum ADCC measured by LDH release after 4 h. Idelalisib activity was assessed (1–1000 nM) in combination with anti-CD20 mAb and normalized to the percentage of maximum cytotoxicity for each donor with Ab only (*n* = 9, in triplicate, mean ± SD). (**B**) The role of FcγRIIIa polymorphisms was assessed in enriched NK cells of the 158 F/F, F/V, and V/V genotypes (*n* = 2 of each) in an ADCC assay, as described above. (**C** and **D**) Human PBMCs were used as effectors with CLL cells as targets at an E:T ratio of 25:1. Anti-CD20 mAb potency was assessed by calcein release after 4 h in the presence of a range of concentrations of idelalisib (1–1000 nM) and a single Ab concentration (10 μg/ml) using obinutuzumab (C) or rituximab (D) (*n* = 4, in triplicate, mean ± SD).

The effect of idelalisib over a range of concentrations (1–1000 nM) was evaluated in ADCC assays using a fixed E:T ratio and saturating amounts of obinutuzumab or rituximab. Idelalisib did not affect rituximab- or obinutuzumab-mediated ADCC in the context of the maximal ADCC activity of each anti-CD20 Ab (Fig. 3A). The impact of the FcγRIIIa genotype was assessed with NK effectors derived from donors with FcγRIIIa 158 phenylalanine homozygous (F/F), phenylalanine/valine (F/V), or valine homozygous (V/V) genotypes. Idelalisib at concentrations up to 1 μM had no significant effect on rituximab- or obinutuzumab-mediated ADCC in FcγRIIIa 158 F/F and F/V genotypes (Fig. 3B). ADCC with the FcγRIIIa 158 V/V effectors was affected slightly by treatment with idelalisib (∼15% inhibition) at the highest concentration tested.

### Impact of idelalisib on ADCC with primary CLL specimens

Next, obinutuzumab- or rituximab-mediated ADCC was assessed in the presence of idelalisib using primary CLL PBMCs as targets. Obinutuzumab and rituximab were effective at inducing ADCC at a saturating concentration of Ab (10 μg/ml), with obinutuzumab inducing 47% ADCC and rituximab inducing 20% ADCC (Fig. 3C, 3D). The amount of ADCC remained largely unchanged for both Abs in combination with idelalisib over a clinically relevant concentration range.

**FIGURE 4.**
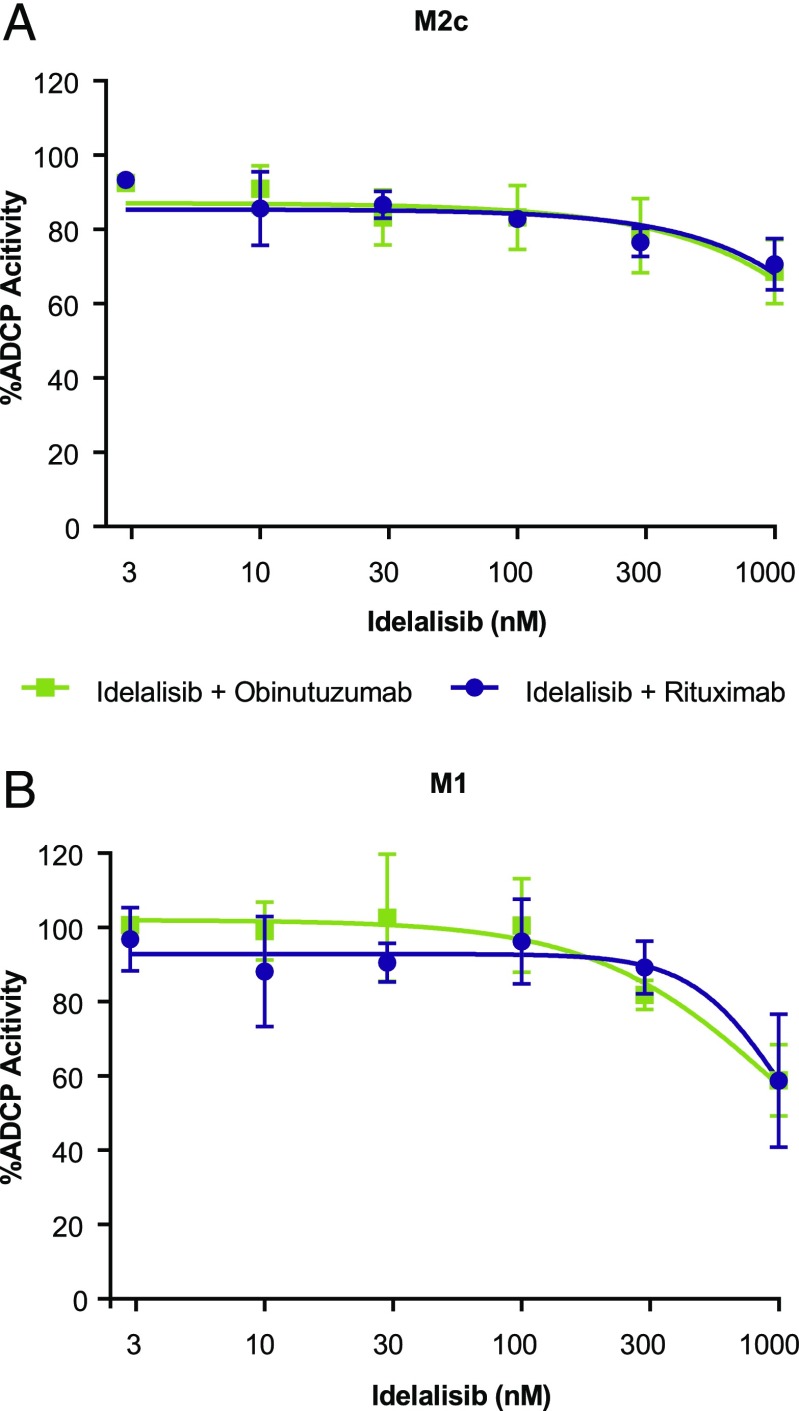
Effect of idelalisib on anti-CD20–mediated ADCP with M2c- and M1-polarized macrophages. MDMs were polarized in vitro toward the M2c phenotype (**A**) or the M1 phenotype (**B**) and used in ADCP assays with the WIL2-S cell line as targets (E:T ratio, 3:1) to evaluate the activity of idelalisib (3–1000 nM) in the presence of anti-CD20 mAb (150 ng/ml). The percentage of phagocytosed target cells was quantified by flow cytometry and normalized to ADCP activity with Ab alone (*n* = 3, in triplicate, mean ± SD).

In addition to ADCC, the effects of idelalisib on obinutuzumab- and rituximab-mediated ADCP were assessed. Primary human MDMs were generated and polarized, and ADCP with obinutuzumab or rituximab was evaluated. ADCP with both Abs was more robust with M2c-directed macrophages (rituximab 61 ± 8%, obinutuzumab 57 ± 14%) than with M1-directed macrophages (rituximab 20 ± 5%, obinutuzumab 20 ± 2%). M2c- and M1-polarized macrophages maintained their ability to effectively phagocytose target cells at up to 300 nM idelalisib, equal to the protein-adjusted C_max_ (Fig. 4).

### Whole-blood Ab-mediated B cell depletion in the presence of idelalisib

**FIGURE 5.**
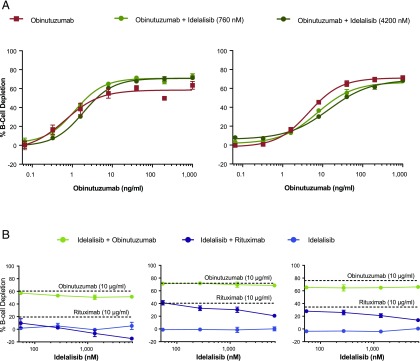
Effect of idelalisib on anti-CD20–mediated whole-blood B cell depletion from healthy volunteers. (**A**) Dose-dependent B cell depletion with obinutuzumab in the presence of clinically relevant concentrations of idelalisib (760 and 4200 nM, minimal concentration and C_max_ in whole blood) in two representative donors (mean ± SEM). (**B**) B cell depletion in the presence of a dose titration of idelalisib (54–6810 nM) in combination with saturating concentrations of obinutuzumab or rituximab (10 μg/ml; the dashed lines represent Ab-alone effect) in three representative donors (mean ± SEM). CD45^+^ cells were gated, and the depletion of B cells (CD19^+^) was calculated by flow cytometry.

The effects of idelalisib in an autologous B cell–depletion assay using a dose titration of obinutuzumab were assessed in the presence of clinically relevant concentrations of idelalisib (760 and 4200 nM, minimal concentration and C_max_ in whole blood). In both donors, B cells were depleted in a dose-dependent manner in the presence of increasing concentrations of obinutuzumab (Fig. 5A). Although there was a slight trend toward inhibition of B cell depletion in one donor, there were no statistically significant effects with idelalisib across the range of obinutuzumab concentrations. Autologous B cell depletion was also tested in the presence of titrated idelalisib (54–6810 nM), in combination with saturating concentrations of obinutuzumab or rituximab, in three donors. B cell depletion by obinutuzumab was largely unaffected by idelalisib (Fig. 5B). In contrast, B cell depletion by rituximab, in combination with idelalisib, varied across donors, with the most inhibition seen at the highest concentration. In all donors tested, idelalisib alone showed no B cell depletion at any concentration.

### Direct induction of cell death in primary CLL specimens

**FIGURE 6.**
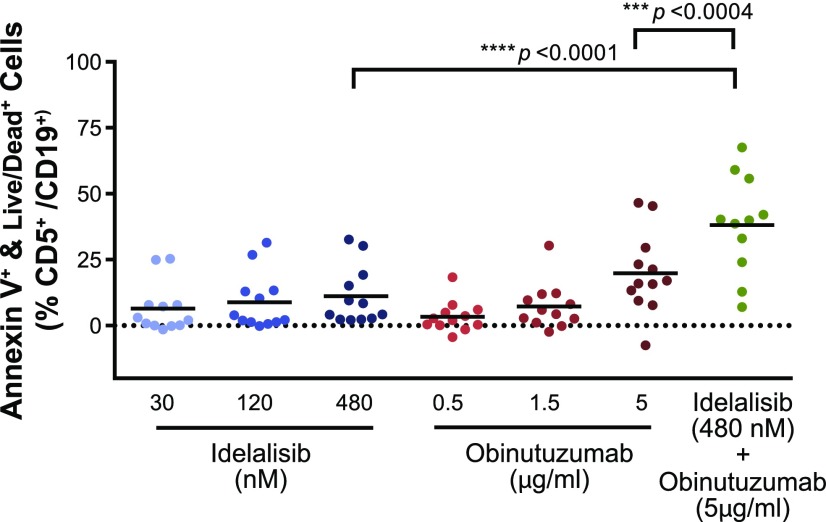
Effect of idelalisib and obinutuzumab on direct cell death induction of CLL cells. Increasing concentrations of idelalisib (30–480 nM) or obinutuzumab (0.5–5 μg/ml) or a combination of 480 nM idelalisib plus 5 μg/ml obinutuzumab were added to CLL cultures stimulated with anti-IgM/IgG/CD40 and incubated for 66 h. CLL cells were gated as CD5^+^/CD19^+^ double-positive and annexin V^+^/viable cells and quantitated by flow cytometry (*n* = 12, in triplicate). Statistical comparisons are pairwise comparisons in the same donor.

Next, we examined the ability of idelalisib, obinutuzumab, or the combination to induce direct cell death in primary CLL cultures stimulated with anti-IgM/IgG/CD40 to mimic prosurvival signaling within the tissues. Idelalisib (30–480 nM) showed a small, but significant, increase in cell death (8–10%) across all doses, whereas obinutuzumab (0.5–5 μg/ml) showed a dose-dependent induction of cell death of 2–21%, which was most prominent at 5 μg/ml (Fig. 6). However, more striking was the ability of the combination of idelalisib (480 nM) and obinutuzumab (5 μg/ml) to elicit a statistically significant increase in cell death (39%) above either agent alone.

### In vivo therapy of Eμ-TCL1–Tg leukemia-bearing mice

**FIGURE 7.**
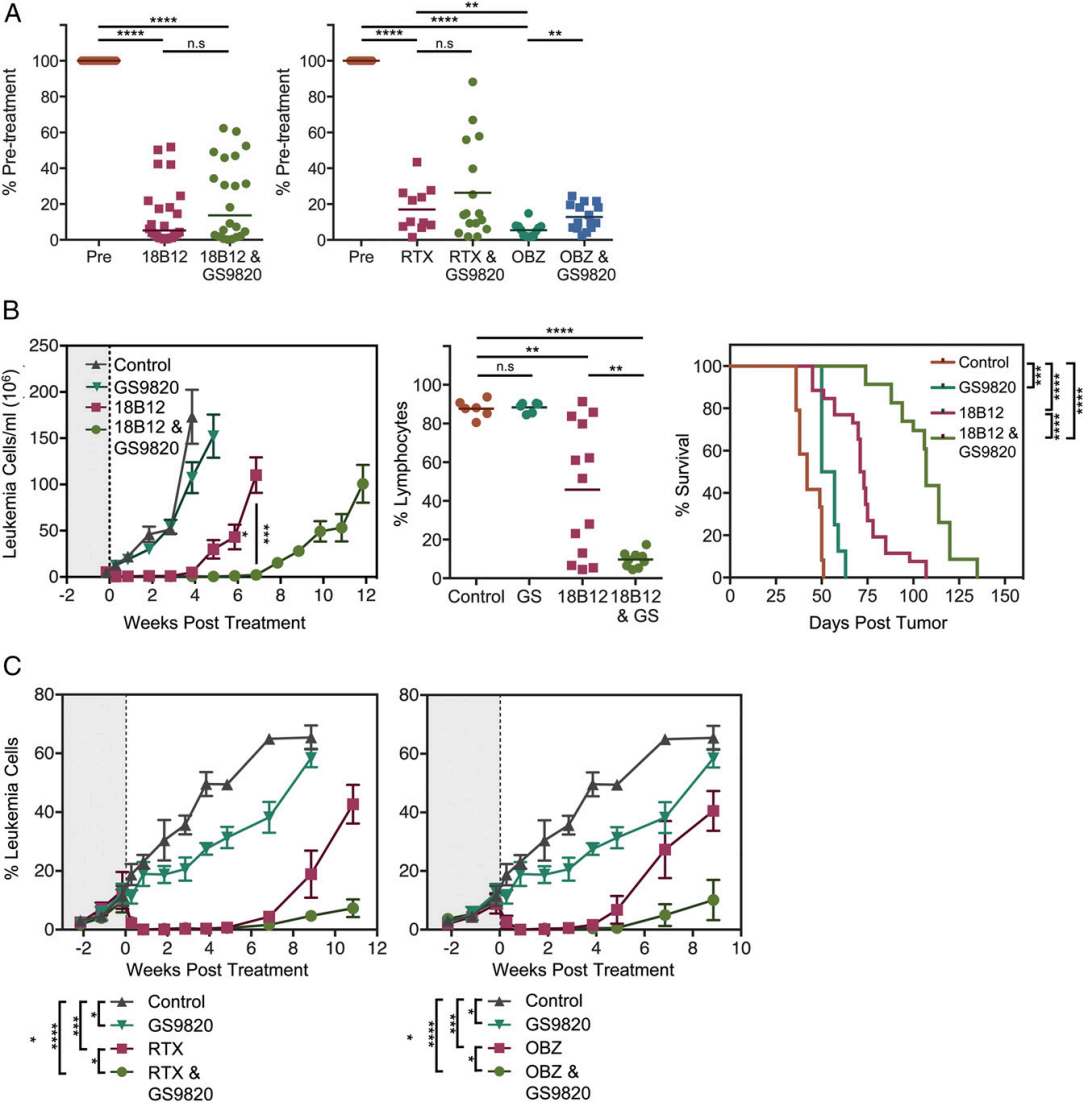
Anti-CD20–mediated depletion of leukemic targets in vivo. (**A**) Animals bearing Eμ-TCL1–Tg (left panel) or human CD20-Tg Eμ-TCL1–Tg leukemia (right panel) were treated with 250 μg of anti-CD20 mAbs (anti-mouse CD20 clone 18B12 mouse IgG2a, rituximab hIgG1 [RTX], or obinutuzumab hIgG1 [OBZ]) alongside 10 mg/kg GS-9820 administered by mouth twice a day or an appropriate vehicle control and were monitored for peripheral leukemia levels 48 h later by flow cytometry. Data are normalized to pretreatment values. (**B**) Eμ-TCL1–Tg tumor (Eμ-TCL1 Tg 020)–bearing animals from (A) were maintained on PI3Kδ inhibition treatment or an appropriate vehicle control for the duration of the experiment and were monitored for leukemia levels by weekly blood sampling and flow cytometry. A representative example of the peripheral leukemic fraction 4 wk posttreatment, expressed as the percentage of total lymphocytes is shown (middle panel). (**C**) Human CD20-Tg Eμ-TCL1–Tg tumor (human CD20–Tg Eμ-TCL1–Tg FU2)–bearing animals from (A) were maintained on PI3Kδ inhibition treatment or an appropriate vehicle control for the duration of the experiment and were monitored for leukemia levels by weekly blood sampling and flow cytometry. Data are represented as the percentage of CD5^+^B220^+^ cells present in the lymphocyte gate. Error bars represent SEM. Statistical analyses were performed using a paired/unpaired Student *t* test (A) and two-way ANOVA or log-rank survival analysis (B and C). *p < 0.05, **p < 0.005, ***p < 0.0005, ****p < 0.000005. GS, GS-9820; n.s, not significant.

We next assessed the impact of PI3Kδ inhibition on the extent and duration of anti-CD20–mediated B cell depletion in a murine model of CLL: the Eμ-TCL1–Tg mouse (22). Because idelalisib is not suitable for use in a murine setting as a result of unfavorable pharmacokinetic properties (21) (S. Tannheimer, Gilead Sciences, unpublished observations), we used the structurally related surrogate PI3Kδ inhibitor, GS-9820. Treatment of leukemia-bearing animals with a suboptimal dose (250 μg per mouse) of anti-mouse CD20 (18B12 mouse IgG2a) or anti-human CD20 mAb (rituximab or obinutuzumab [250 μg of each hIgG_1_ per mouse]) imparted a rapid and significant reduction in the leukemic burden of treated animals 48 h posttreatment (Fig. 7A). Consistent with previous studies, initial depletion of target cells with obinutuzumab was greater than that achieved with rituximab (*p* < 0.005). In this model system, concomitant PI3Kδ inhibition (via GS-9820) induced a statistically significant reduction in the extent of obinutuzumab-mediated depletion and a trend toward reduction in the rituximab and 18B12 treatment settings. In a monotherapy setting, GS-9820 provided a modest therapeutic benefit and effectively reduced the extent of leukemia deposits within secondary lymphoid organs (21) (Fig. 7B, 7C, Supplemental Fig. 1). Therefore, the apparent reduction in anti-CD20–mediated depletion 48 h posttreatment in the presence of PI3Kδ inhibition may reflect a redistribution of target cells into the periphery, as is observed in clinical trials (23), rather than inhibition of Ab effector mechanisms. In support of this, concomitant administration of a PI3Kδ inhibitor with a single dose of anti-mouse or anti-human CD20 mAb provided a more durable depletion of leukemic cells over the long term and significantly enhanced overall survival in comparison with Ab therapy alone (Fig. 7B, 7C). Although obinutuzumab appeared to outperform rituximab in a short-term depletion setting, long-term superiority of one agent over the other was observed between different Eμ-TCL1–Tg tumors (Supplemental Fig. 1). This observation is most likely attributable to the suboptimal nature of the Ab-dosing strategy. These data show that, despite the marginal impact of PI3Kδ inhibition on both in vitro Ab effector mechanism assays and in vivo depletion in the first 48 h with obinutuzumab, these combinations provide additional benefit to the duration of leukemic depletion in a therapeutic model.

## Discussion

The recent regulatory approvals of multiple new targeted therapies, including the anti-CD20 mAb, obinutuzumab, have provided many treatment options for patients with CLL. Multiple small molecule inhibitors that target the BCR pathway have been approved for use in CLL, with idelalisib approved for use with rituximab in relapsed and refractory CLL (4, 5). A key step in the clinical integration of these therapies is a clear understanding of how these agents might be combined. Our studies evaluated the effect of idelalisib (or its surrogate GS-9820) at physiologically relevant concentrations on direct killing, ADCC, ADCP, and in vivo therapy in combination with rituximab or obinutuzumab. Rituximab is a type I Ab, whereas obinutuzumab is a type II glycoengineered Ab that has enhanced affinity for FcγRIIIa on effector cells, which leads to enhanced ADCC (11, 24). Differences between the two classes of Abs have also been shown for direct cell killing (9, 25), complement-dependent cytotoxicity (6), and binding-induced CD20 internalization (26), but they seem to have comparable effects on ADCP (10, 27, 28).

Our results using obinutuzumab and rituximab in ADCC assays corroborate previous studies showing that obinutuzumab results in better outcomes, regardless of the CD20 levels of the target, Ab concentration, or E:T ratio used in the assay (10). Our studies showed that idelalisib had no effect on anti-CD20 ADCC with obinutuzumab or rituximab, in contrast to other studies that used higher concentrations than those in the clinic (29, 30). Previous reports have shown that the F/V polymorphism of FcγRIIIa can impact NK cell binding to Ab (31), as well as progression-free survival, when evaluated in the context of rituximab treatment in genotyped patients (32).

When the impact of idelalisib was assessed in vitro on NK cells with known FcγRIIIa polymorphisms, it showed no inhibition of ADCC, regardless of genotype. Our findings also demonstrated that F/F and F/V polymorphisms were no less effective in ADCC cell assays with obinutuzumab. Characterization of NK cells from PI3Kδ kinase–dead or -knockout mice shows altered NK cell migration, extravasation, receptor activation, and cytokine production, although alterations in cytotoxicity toward tumor cells in vitro or in vivo was not observed (33–35). In our in vitro studies investigating the effects of idelalisib on NK cell–mediated ADCC, we saw a lack of inhibition of NK cell function, as well as observed that CD20 levels on target cells are not altered by PI3Kδ inhibition (data not shown). Additionally, unlike ibrutinib, which has been shown to suppress ADCC and ADCP function due to strong inhibition of IL-2–inducible tyrosine kinase, idelalisib has no activity on Bruton’s tyrosine kinase or IL-2–inducible tyrosine kinase (16, 36, 37).

MDMs, polarized to M2c or M1, showed robust phagocytosis of target cells, with M2c demonstrating superior activity. Idelalisib had no effect on macrophage ADCP function on either subset up to the protein-adjusted C_max_ ∼ 300 nM. The robust phagocytosis of myeloid cells in this in vitro assay suggests that idelalisib treatment has little to no impact on macrophage function. More significantly, there is no effect of idelalisib on anti-CD20–mediated B cell depletion from whole blood in healthy donors, an assay that may best model the clinical experience as a result of the integration of multiple mechanisms for depletion (i.e., ADCC, complement-dependent cytotoxicity, induction of cell death). In agreement with these findings, administration of a surrogate PI3Kδ inhibitor (GS-9820) alongside suboptimal doses of anti-mouse CD20, rituximab, or obinutuzumab only minimally influenced maximal depletion of leukemic targets in a murine model of CLL in vivo. These observations indicate that key Ab effector mechanisms are not significantly affected in vivo by PI3Kδ inhibition, and any apparent reduction in anti-CD20–mediated depletion in the presence of PI3Kδ inhibition may reflect a redistribution of target cells into the periphery, as is observed in clinical trials (23). More significantly, in this model, administration of PI3Kδ inhibition alongside anti-CD20 mAbs imparted a more durable depletion of leukemic targets and enhanced therapeutic effects. These observations are reminiscent of clinical trial results demonstrating the therapeutic enhancement of rituximab therapy offered by coadministration of idelalisib in relapsed CLL patients (23).

When the combination of idelalisib and obinutuzumab was evaluated in terms of cell death induction of primary donor CLL cells, a strong additive antitumor activity was confirmed. This unexpected enhanced activity, in conjunction with the lack of idelalisib inhibition of obinutuzumab-mediated ADCC and ADCP activity, suggests that a glycoengineered type II anti-CD20 Ab may function as effectively as a type I Ab in combination with idelalisib.

In conclusion, at clinically relevant doses, idelalisib does not have significant effects on rituximab- or obinutuzumab-driven ADCC, ADCP, or B cell–depletion activity. Additionally, the combination of obinutuzumab and idelalisib appears to have additive activities on direct killing of CLL cells and control of leukemic burden in vivo compared with either agent alone. These studies provide preclinical rationale that the addition of idelalisib to type II Abs, such as obinutuzumab, is likely to be effective clinically without negatively impacting any Ab-mediated immune functions. Based on these findings, obinutuzumab is being tested in combination with idelalisib in clinical trials (NCT02962401, NCT02968563, NCT02445131).

## Supplementary Material

Data Supplement

## References

[1] Lannutti B. J. Meadows S. A. Herman S. E. M. Kashishian A. Steiner B. Johnson A. J. Byrd J. C. Tyner J. W. Loriaux M. M. Deininger M. 2011 CAL-101, a p110δ selective phosphatidylinositol-3-kinase inhibitor for the treatment of B-cell malignancies, inhibits PI3K signaling and cellular viability. Blood 117: 591–594. 20959606 10.1182/blood-2010-03-275305 PMC3694505

[2] Puri K. D. Gold M. R. 2012 Selective inhibitors of phosphoinositide 3-kinase delta: modulators of B-cell function with potential for treating autoimmune inflammatory diseases and B-cell malignancies. Front Immunol. 3: 256. 22936933 10.3389/fimmu.2012.00256 PMC3425960

[3] Vanhaesebroeck B. Guillermet-Guibert J. Graupera M. Bilanges B. 2010 The emerging mechanisms of isoform-specific PI3K signalling. Nat. Rev. Mol. Cell Biol. 11: 329–341. 20379207 10.1038/nrm2882

[4] ZYDELIG (idelalisib) tablets, for oral use initial U.S. approval: 2014 http://www.gilead.com/∼/media/Files/pdfs/medicines/oncology/zydelig/zydelig_pi.pdf. Accessed: December 1, 2017.

[5] Zydelig (idelalisib) tablets, for oral use initial E.U. approval: 2014 Available at: http://www.ema.europa.eu/ema/index.jsp?curl=pages/medicines/human/medicines/003843/human_med_001803.jsp&mid=WC0b01ac058001d124. Accessed: December 1, 2017.

[6] Cragg M. S. Morgan S. M. Chan H. T. Morgan B. P. Filatov A. V. Johnson P. W. M. French R. R. Glennie M. J. 2003 Complement-mediated lysis by anti-CD20 mAb correlates with segregation into lipid rafts. Blood 101: 1045–1052. 12393541 10.1182/blood-2002-06-1761

[7] Cragg M. S. Glennie M. J. 2004 Antibody specificity controls in vivo effector mechanisms of anti-CD20 reagents. Blood 103: 2738–2743. 14551143 10.1182/blood-2003-06-2031

[8] Deans J. P. Robbins S. M. Polyak M. J. Savage J. A. 1998 Rapid redistribution of CD20 to a low density detergent-insoluble membrane compartment. J. Biol. Chem. 273: 344–348. 9417086 10.1074/jbc.273.1.344

[9] Ivanov A. Beers S. A. Walshe C. A. Honeychurch J. Alduaij W. Cox K. L. Potter K. N. Murray S. Chan C. H. T. Klymenko T. 2009 Monoclonal antibodies directed to CD20 and HLA-DR can elicit homotypic adhesion followed by lysosome-mediated cell death in human lymphoma and leukemia cells. J. Clin. Invest. 119: 2143–2159. 19620786 10.1172/JCI37884 PMC2719942

[10] Herter S. Herting F. Mundigl O. Waldhauer I. Weinzierl T. Fauti T. Muth G. Ziegler-Landesberger D. Van Puijenbroek E. Lang S. 2013 Preclinical activity of the type II CD20 antibody GA101 (obinutuzumab) compared with rituximab and ofatumumab in vitro and in xenograft models. Mol. Cancer Ther. 12: 2031–2042. 23873847 10.1158/1535-7163.MCT-12-1182

[11] Mössner E. Brünker P. Moser S. Püntener U. Schmidt C. Herter S. Grau R. Gerdes C. Nopora A. van Puijenbroek E. 2010 Increasing the efficacy of CD20 antibody therapy through the engineering of a new type II anti-CD20 antibody with enhanced direct and immune effector cell-mediated B-cell cytotoxicity. Blood 115: 4393–4402. 20194898 10.1182/blood-2009-06-225979 PMC2881503

[12] GAZYVA (obinutuzumab) injection, for intravenous use initial U.S. approval: 2013 https://www.accessdata.fda.gov/drugsatfda_docs/label/2016/125486s013lbl.pdf. Accessed: December 1, 2017.

[13] Gazyvaro (obinutuzumab) injection, for intravenous use initial E.U. approval: 2014 http://www.ema.europa.eu/ema/index.jsp?curl=pages/medicines/human/medicines/002799/human_med_001780.jsp&mid=WC0b01ac058001d124. Accessed: December 1, 2017.

[14] Marcus R. Davies A. Ando K. Klapper W. Opat S. Owen C. Phillips E. Sangha R. Schlag R. Seymour J. F. 2017 Obinutuzumab for the first-line treatment of follicular lymphoma. N. Engl. J. Med. 377: 1331–1344. 28976863 10.1056/NEJMoa1614598

[15] Sehn L. H. Chua N. Mayer J. Dueck G. Trněný M. Bouabdallah K. Fowler N. Delwail V. Press O. Salles G. 2016 Obinutuzumab plus bendamustine versus bendamustine monotherapy in patients with rituximab-refractory indolent non-Hodgkin lymphoma (GADOLIN): a randomised, controlled, open-label, multicentre, phase 3 trial. Lancet Oncol. 17: 1081–1093. 27345636 10.1016/S1470-2045(16)30097-3

[16] Vitolo U. Trněný M. Belada D. Burke J. M. Carella A. M. Chua N. Abrisqueta P. Demeter J. Flinn I. Hong X. 2017 Obinutuzumab or rituximab plus cyclophosphamide, doxorubicin, vincristine, and prednisone in previously untreated diffuse large b-cell lymphoma. J. Clin. Oncol. 35: 3529–3537. 28796588 10.1200/JCO.2017.73.3402

[17] Vitolo U. Trněný M. Belada D. Carella A. M. Chua N. Abrisqueta P. Demeter J. Flinn I. W. Hong X. Kin W. S. Obinutuzumab or rituximab plus CHOP in patients with previously untreated diffuse large B-cell lymphoma: final results from an open-label, randomized phase 3 study (GOYA). Abstract presented at ASH 58th Annual Meeting & Exposition. December 3–6, 2016; San Diego, CA. (Abstr. 470).

[18] Kohrt H. E. Sagiv-Barfi I. Rafiq S. Herman S. E. Butchar J. P. Cheney C. Zhang X. Buggy J. J. Muthusamy N. Levy R. 2014 Ibrutinib antagonizes rituximab-dependent NK cell-mediated cytotoxicity. Blood 123: 1957–1960. 24652965 10.1182/blood-2014-01-547869 PMC3962169

[19] Okkenhaug K. 2013 Signaling by the phosphoinositide 3-kinase family in immune cells. Annu. Rev. Immunol. 31: 675–704. 23330955 10.1146/annurev-immunol-032712-095946 PMC4516760

[20] Ahuja A. Shupe J. Dunn R. Kashgarian M. Kehry M. R. Shlomchik M. J. 2007 Depletion of B cells in murine lupus: efficacy and resistance. J. Immunol. 179: 3351–3361. 17709552 10.4049/jimmunol.179.5.3351

[21] Carter M. J. Cox K. L. Blakemore S. J. Turaj A. H. Oldham R. J. Dahal L. N. Tannheimer S. Forconi F. Packman G. Cragg M. S. 2017 PI3Kδ inhibition elicits anti-leukemic effects through Bim-dependent apoptosis. Leukemia 31: 1423–1433. 27843137 10.1038/leu.2016.333 PMC5467045

[22] Bichi R. Shinton S. A. Martin E. S. Koval A. Calin G. A. Cesari R. Russo G. Hardy R. R. Croce C. M. 2002 Human chronic lymphocytic leukemia modeled in mouse by targeted TCL1 expression. Proc. Natl. Acad. Sci. USA 99: 6955–6960. 12011454 10.1073/pnas.102181599 PMC124510

[23] Furman R. R. Sharman J. P. Coutre S. E. Cheson B. D. Pagel J. M. Hillmen P. Barrientos J. C. Zelenetz A. D. Kipps T. J. Flinn I. 2014 Idelalisib and rituximab in relapsed chronic lymphocytic leukemia. N. Engl. J. Med. 370: 997–1007. 24450857 10.1056/NEJMoa1315226 PMC4161365

[24] Ferrara C. Grau S. Jäger C. Sondermann P. Brünker P. Waldhauer I. Hennig M. Ruf A. Rufer A. C. Stihle M. 2011 Unique carbohydrate-carbohydrate interactions are required for high affinity binding between FcgammaRIII and antibodies lacking core fucose. Proc. Natl. Acad. Sci. USA 108: 12669–12674. 21768335 10.1073/pnas.1108455108 PMC3150898

[25] Chan H. T. Hughes D. French R. R. Tutt A. L. Walshe C. A. Teeling J. L. Glennie M. J. Cragg M. S. 2003 CD20-induced lymphoma cell death is independent of both caspases and its redistribution into triton X-100 insoluble membrane rafts. Cancer Res. 63: 5480–5489. 14500384

[26] Beers S. A. French R. R. Chan H. T. C. Lim S. H. Jarrett T. C. Vidal R. M. Wijayaweera S. S. Dixon S. V. Kim H. Cox K. L. 2010 Antigenic modulation limits the efficacy of anti-CD20 antibodies: implications for antibody selection. Blood 115: 5191–5201. 20223920 10.1182/blood-2010-01-263533

[27] Bologna L. Gotti E. Manganini M. Rambaldi A. Intermesoli T. Introna M. Golay J. 2011 Mechanism of action of type II, glycoengineered, anti-CD20 monoclonal antibody GA101 in B-chronic lymphocytic leukemia whole blood assays in comparison with rituximab and alemtuzumab. J. Immunol. 186: 3762–3769. 21296976 10.4049/jimmunol.1000303

[28] Rafiq S. Butchar J. P. Cheney C. Mo X. Trotta R. Caligiuri M. Jarjoura D. Tridandapani S. Muthusamy N. Byrd J. C. 2013 Comparative assessment of clinically utilized CD20-directed antibodies in chronic lymphocytic leukemia cells reveals divergent NK cell, monocyte, and macrophage properties. J. Immunol. 190: 2702–2711. 23418626 10.4049/jimmunol.1202588 PMC3631574

[29] Da Roit F. Engelberts P. J. Taylor R. P. Breij E. C. Gritti G. Rambaldi A. Introna M. Parren P. W. Beurskens F. J. Golay J. 2015 Ibrutinib interferes with the cell-mediated anti-tumor activities of therapeutic CD20 antibodies: implications for combination therapy. Haematologica 100: 77–86. 25344523 10.3324/haematol.2014.107011 PMC4281316

[30] Duong M. N. Matera E.-L. Mathé D. Evesque A. Valsesia-Wittmann S. Clémenceau B. Dumontet C. 2015 Effect of kinase inhibitors on the therapeutic properties of monoclonal antibodies. MAbs 7: 192–198. 25523586 10.4161/19420862.2015.989020 PMC4622495

[31] Koene H. R. Kleijer M. Algra J. Roos D. von dem Borne A. E. de Haas M. 1997 Fc gammaRIIIa-158V/F polymorphism influences the binding of IgG by natural killer cell Fc gammaRIIIa, independently of the Fc gammaRIIIa-48L/R/H phenotype. Blood 90: 1109–1114. 9242542

[32] Cartron G. Dacheux L. Salles G. Solal-Celigny P. Bardos P. Colombat P. Watier H. 2002 Therapeutic activity of humanized anti-CD20 monoclonal antibody and polymorphism in IgG Fc receptor FcgammaRIIIa gene. Blood 99: 754–758. 11806974 10.1182/blood.v99.3.754

[33] Guo H. Samarakoon A. Vanhaesebroeck B. Malarkannan S. 2008 The p110 δ of PI3K plays a critical role in NK cell terminal maturation and cytokine/chemokine generation. J. Exp. Med. 205: 2419–2435. 18809712 10.1084/jem.20072327 PMC2556795

[34] Saudemont A. Garçon F. Yadi H. Roche-Molina M. Kim N. Segonds-Pichon A. Martín-Fontecha A. Okkenhaug K. Colucci F. 2009 p110γ and p110δ isoforms of phosphoinositide 3-kinase differentially regulate natural killer cell migration in health and disease. Proc. Natl. Acad. Sci. USA 106: 5795–5800. 19297623 10.1073/pnas.0808594106 PMC2667007

[35] Kim N. Saudemont A. Webb L. Camps M. Ruckle T. Hirsch E. Turner M. Colucci F. 2007 The p110delta catalytic isoform of PI3K is a key player in NK-cell development and cytokine secretion. Blood 110: 3202–3208. 17644738 10.1182/blood-2007-02-075366

[36] Khurana D. Arneson L. N. Schoon R. A. Dick C. J. Leibson P. J. 2007 Differential regulation of human NK cell-mediated cytotoxicity by the tyrosine kinase Itk. J. Immunol. 178: 3575–3582. 17339454 10.4049/jimmunol.178.6.3575

[37] Somoza J. R. Koditek D. Villaseñor A. G. Novikov N. Wong M. H. Liclican A. Xing W. Lagpacan L. Wang R. Schultz B. E. 2015 Structural, biochemical, and biophysical characterization of idelalisib binding to phosphoinositide 3-kinase δ. J. Biol. Chem. 290: 8439–8446. 25631052 10.1074/jbc.M114.634683 PMC4375495

