# Supplementary material for: The PI3Kδ-Selective Inhibitor Idelalisib Minimally Interferes with Immune Effector Function Mediated by Rituximab or Obinutuzumab and Significantly Augments B Cell Depletion In Vivo
Source: J Immunol. 2018 Feb 16;200(7):2304–12. doi: 10.4049/jimmunol.1700323 (PMC5857647; doi:10.4049/jimmunol.1700323)
Supplement: Data Supplement [file JI_1700323.zip › JI_1700323_Supplemental_Figure_1.pdf]

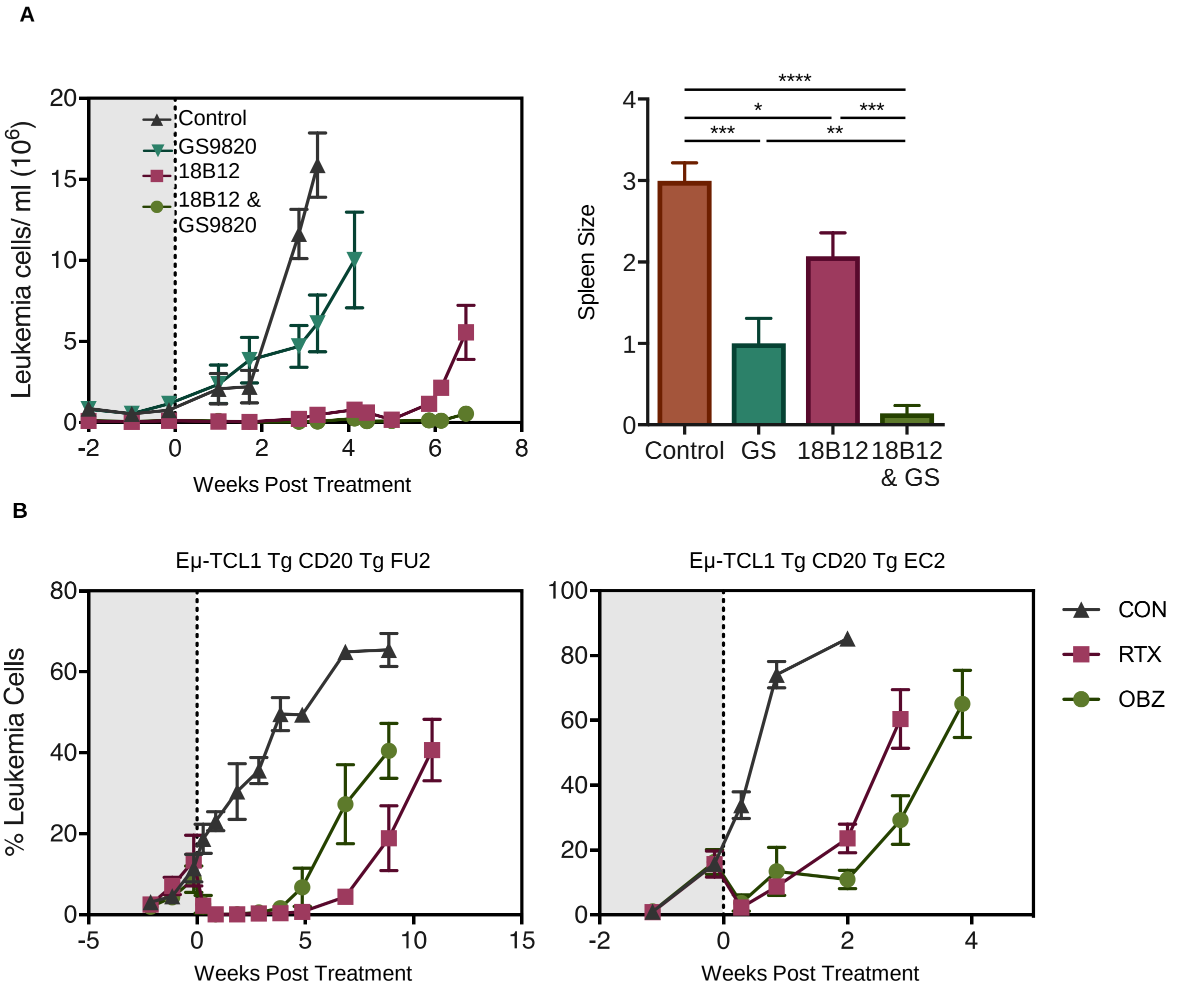

Supplemental Figure 1. Tumor tissue deposits and the relative efficacy of rituximab and obinutuzumab. **(A)** Animals bearing an alternate Eμ-TCL1 Tg leukemia (Eμ-TCL1 Tg U3) were treated with 250 μg anti-mouse CD20 alongside 10 mg/kg GS-9820 administered per os BID or an appropriate vehicle control and monitored for peripheral leukemia levels by blood sampling and flow cytometry (shown left). Splenomegaly was assessed by palpation 3 weeks post treatment and scored by an arbitrary system, in which a non-tumor bearing C57BL/6 is scored as 0 and a tumor bearing spleen 1 inch in length scored as 3. GS denotes GS9820. **(B)** Animals bearing different hCD20Tg Eμ-TCL1 Tg tumors (either FU2 or EC2) were assessed for the relative efficacy of rituximab (RTX) or obinutuzumab (OBZ) therapy (both hIgG1). Animals were treated with 250 μg RTX or OBZ once leukemias were detectable in the blood and monitored for disease progression by blood sampling and flow cytometry. Error bars represent SEM. Statistical analysis was performed using an un-paired Student's T test.

* = $p < 0.05$, ** = $p < 0.005$, *** = $p < 0.0005$, **** = $p < 0.00005$.
